# MicroRNAs: Their Role in Metabolism, Tumor Microenvironment, and Therapeutic Implications in Head and Neck Squamous Cell Carcinoma

**DOI:** 10.3390/cancers13225604

**Published:** 2021-11-09

**Authors:** Shine-Gwo Shiah, Sung-Tau Chou, Jang-Yang Chang

**Affiliations:** 1 National Institute of Cancer Research, National Health Research Institutes, Miaoli 35053, Taiwan; davidssg@nhri.edu.tw (S.-G.S.); 014050@nhri.edu.tw (S.-T.C.); 2 Cancer Center, Wan Fang Hospital, Taipei Medical University, Taipei 11031, Taiwan; 3 Institute of Biotechnology and Pharmaceutical Research, National Health Research Institutes, Miaoli 35053, Taiwan

**Keywords:** miRNA, HNSCC, metabolism, exosomes, tumor microenvironment

## Abstract

**Simple Summary:**

Head and neck squamous cell carcinoma (HNSCC), which arises from the oral epithelium, is one of the most common cancers worldwide. Despite excellent diagnosis and treatment improvements, the mortality rate associated with HNSCC is still extremely high. Current data suggest that dysregulation of exosomes and metabolic abnormalities are involved in the initiation and progression of HNSCC. Thus, approaches for targeting exosomes in the tumor microenvironment and metabolic reprogramming pathways represent potential therapeutic strategies. Moreover, some miRNAs are thought to have significant functions in regulating the progression of HNSCC. The present article aims to summarize the current knowledge concerning the important miRNAs in both exosomes and cancer metabolism, as well as discuss future perspectives regarding their future diagnostic potential and treatment recommendations.

**Abstract:**

MicroRNAs (miRNAs) are endogenous small non-coding RNA molecules that negatively regulate gene expression by binding to target mRNAs. Deregulated miRNAs can act as either oncogenic miRNAs or tumor suppressor miRNAs in controlling proliferation, differentiation, apoptosis, metastasis, epithelial–mesenchymal transition, and immune responses, which are all involved in the carcinogenesis process of HNSCC. Recent findings have shown that metabolic reprogramming is an important hallmark of cancer, which is necessary for malignant transformation and tumor development. Some reprogrammed metabolisms are believed to be required for HNSCC against an unfavorable tumor microenvironment (TME). The TME is composed of various cell types embedded in the altered extracellular matrix, among which exosomes, secreted by cancer cells, are one of the most important factors. Tumor-derived exosomes reshape the tumor microenvironment and play a crucial role in cell-to-cell communication during HNSCC development. Exosomes encapsulate many biomolecules, including miRNAs, circulate in body fluids, and can transmit intercellular regulatory messages to nearby and distant sites, which indicates that exosomal miRNAs have the potential to become non-invasive biomarkers. This review aims to clarify the functions of diverse miRNAs in HNSCC metabolic reprogramming and tumor-derived exosomes. In addition, it also emphasizes the potential role of miRNA as a biomarker in the diagnosis, prognosis, and treatment of HNSCC cancer.

## 1. Introduction

Head and neck squamous cell carcinoma (HNSCC), which develops from the oral cavity, oropharynx, larynx, or hypopharynx, is the sixth most common malignancy in the world [1,2]. Epidemiological analysis shows that alcohol consumption, tobacco exposure, and human papillomavirus (HPV) infection are several known high risk factors for HNSCC [3]. Additionally, among some Asia-Pacific populations, areca nut or betel quid products have been recognized as a major contributing factor for oral squamous cell carcinoma (OSCC) incidence and mortality rates [1,4]. Despite there being multiple therapy options for HNSCC, including surgery, chemotherapy, radiotherapy, and immunotherapy, approximately half of treated patients die within 5 years of diagnosis because patients still often have locoregional recurrences, second primary cancers, and distant metastases [5]. Additionally, intrinsic and extrinsic resistance to chemotherapy and radiation therapy are also major obstacles of failed cancer therapy, and they contribute to poor prognosis of HNSCC patients [6]. As such, there is an urgent need to find effective prognostic and predictive biomarkers, associated with the clinical prognosis of HNSCC, which lead to an improvement in the overall survival of HNSCC.

In the past two decades, massive next generation sequencing (NGS) and transcriptome profiling have provided much information for screening and searching for new cancer genes [7]. Among these studies, microRNAs (miRNAs) have been identified as excellent biomarkers in the diagnosis of human diseases, including HNSCC [8,9,10,11]. In addition to their high tissue specificity, the main reason why miRNAs are ideal cancer biomarkers is their high stability in body fluids (including blood, urine, and saliva), even in paraffin-embedded specimens [12]. These criteria are necessary for a quick and accurate diagnostic process. Several studies have successfully applied miRNA expression signatures to determine cancer identification, diagnostic potential and treatment prognosis [9,13,14,15], even with higher accuracy than using mRNA expression profiles [16]. In terms of molecular structure evolution, miRNAs are endogenous small non-coding RNA molecules, containing about 18–24 nucleotides that negatively regulate gene expression by binding to the 3′-untranslated regions (3′-UTR) of target mRNAs [17,18]. Due to the diversity of its binding site and imperfect matching to the target sequence, one miRNA can target multiple mRNAs [19]. Based on this characteristic of multiple-target gene regulation, miRNAs have key roles in most cancer hallmarks, such as proliferation, differentiation, apoptosis, recurrence, metastasis, epithelial–mesenchymal transition (EMT), and immune response [20,21]. Increasing evidence has shown that deregulated miRNAs can act as either oncogenic miRNAs or tumor suppressor miRNAs in controlling these signaling pathways, which are involved in the carcinogenesis process of HNSCC [22,23,24,25,26].

Recently, cancer metabolic pathways, in HNSCC and its surrounding microenvironment, have attracted increasing attention. Metabolic reprogramming is regulated through a complex network in cells, where energy production is switched from oxidative phosphorylation (OXPHOS) to glycolysis [27]. Some reprogrammed metabolisms are believed to be required for cancer cells to respond to a variety of intrinsic and extrinsic changes [28]. The tumor microenvironment (TME) is a combination of various molecules derived from cancer cells and surrounding stroma cells. One of the most important factors of the TME is exosomes. Tumor-derived exosomes reshape the TME and play a crucial role in cell-to-cell communication during tumor development [29]. In this review, we place emphasis on the roles of miRNAs in HNSCC metabolic reprogramming and in tumor-derived exosomes, with particular attention directed towards providing new approaches for HNSCC therapeutic applications.

## 2. miRNAs in HNSCC Metabolism

Cancer metabolic reprogramming is a highly dynamic evolutionary process that allows cancer cells to adapt to environmental challenges and facilitates the transformation of a cell to a malignant phenotype. Aerobic glycolysis (the Warburg effect) is the first discovered and the most well-known altered metabolic pathway; it is a cellular status, favored in cancer cells, in which energy production is less efficient but is characterized by quick generation for cancer requirements. During the transition from OXPHOS to glycolysis, the glycolytic intermediates can be used in the biosynthesis of the macromolecules needed for mitochondrial metabolism, lipid synthesis, amino acid metabolism, and nucleotide production [30]. Some reprogrammed metabolisms have been identified as supporting the needs of rapid cell growth, survival in harsh conditions, migration, invasion, and resistance to drug treatments [31], as well as allowing a rapid adaptation to the microenvironment. Growing evidence has shown that miRNAs can directly or indirectly affect the changes to metabolic enzymes that mediate metabolic reprogramming in HNSCC [32]. A summary of miRNAs, their key targets, and their associated applications involved in HNSCC metabolism are shown in Table 1.

### 2.1. miR-218, miR-10a, miR-340

Cancer cells need to increase nutrient intake to support a high proliferation rate. Experimental data from positron emission tomography (PET) and computed tomography (CT) show that HNSCC requires substantial amounts of energy supply and has a higher glucose intake rate [40]. Obviously, the glucose transporter (GLUT) proteins play an important role in glucose transport in cancer cells. The GLUT family consists of 14 members; however, GLUT1 and GLUT3 are the two most common forms aberrantly expressed in OSCC, facilitating the maintenance of glycolytic energy metabolism [41]. MiR-218 is a tumor-suppressive miRNA that is usually downregulated and plays its biological role by regulating the proliferation migration, invasion, and metastasis of certain types of cancers [42], including OSCC [33]. Xu et al. demonstrated that inhibition of miR-218 can promote oral cancer cell growth by targeting GLUT1 to affect glucose metabolism. Furthermore, serum miR-218 and GLUT1 can serve as effective and accurate biomarkers for the diagnosis and prognosis of oral cancer patients [33]. Similar to miR-218, miR-340 has also been reported to down-regulate in OSCC and work as a metabolic switch by regulating GLUT1 expression, leading to an increase in the glucose absorption rate and lactate secretion [34]. Another miRNA of interest is miR-10a, which may play an oncogenic role in oral cancer by upregulating GLUT1 and increasing glucose metabolism [35]. Because there is no potential binding site of miR-10a in the 3′-UTR of GLUT1 mRNA, miR-10a may not be able to directly target GLUT1 [35]. These data suggest that miR-10a may act as an upstream regulator of GLUT1 in OSCC for promoting cancer cell proliferation and glucose uptake.

### 2.2. miR-143, miR-125-b-5p

Hexokinase (HK) converts glucose to glucose-6-phosphate, which is the initial rate-limiting step in the glycolytic metabolic pathway and a key step in the direction of glucose flux into the cells [43]. To date, four HK isoforms (HK1-4) have been characterized in mammalian tissues [44]. In clinical studies, a high level of HK2 has been detected in various cancers and is associated with poor prognosis and overall survival [45,46]. In HNSCC cells, HK2 has also been proven to drive metabolic reprogramming towards OXPHOS. Furthermore, HK2 is a major promoter of carcinogenesis by regulating the signals mediated by oncogenic Akt and mutant TP53 in HNSCC cells [47]. Recently, Sun et al. reported that miR-143 was remarkably downregulated in oral cancer patient specimens and targeted the 3′-UTR of HK2 [36]. Overexpression of miR-143 causes the downregulation of HK2 protein in oral cancer, leading to a reduction in glucose consumption. The reduction in glucose uptake will result in significant inhibition of proliferation, migration, and invasion. Another case of a tumor-suppressive miRNA is miR-125b-5p, which has been found to be downregulated in laryngeal squamous cell carcinoma (LSCC) [37]. Similar to the response of miR-143, the upregulation of miR-125b-5p also negatively regulates the expression of HK2 to reduce glucose consumption and lactate production, thereby inducing growth inhibition of LSCC cells.

### 2.3. miR-210

Hypoxia refers to oxygen deprivation, and this frequently occurs in many different cancer types, stimulating complex oncogenic signaling in tumors to support tumor cells’ proliferation, survival, malignant progression, and metabolic activity [48]. These broad tumor phenotypes are coordinated by a hypoxia-inducible factor (HIF), which transcriptionally regulates downstream genes in response to hypoxic environments, and especially functions as a key regulator of metabolic reprogramming [49]. For example, hypoxia induces genes involved in extracellular pH regulation, including monocarboxylate transporter 4 (MCT4) and sodium-hydrogen exchanger 1 (NHE1), as well as carbonic anhydrase 9 (CA9), which pump lactate and H+ ions out of cancer cells to maintain intracellular alkalinization and extracellular acidification [50]. Recently, it has been found that the number of miRNAs regulated by HIF-1α mediates changes in cell metabolism [51], and the most studied hypoxia-induced miRNA is miR-210 [52,53]. HIF-1α-induced miR-210 is observed in many cell types, and it has been described as being involved in broad cancer pathologies, including DNA repair [54], survival [55], proliferation [56], metastasis [57], epithelial–mesenchymal transition (EMT) [58], angiogenesis [59], and radioresistance [60]. In oropharyngeal squamous cell carcinomas (OPSCC), iron-sulfur cluster assembly protein (ISCU) has been confirmed as a target of the HIF-1α-induced miR-210 [38]. ISCU facilitates the assembly of iron-sulfur clusters into the enzymes, which is helpful for energy production through the TCA cycle, and contributes to the activity of mitochondrial respiratory complexes [61]. Therefore, miR-210-mediated ISCU destruction facilitates a switch from mitochondrial respiration to glycolytic metabolism, which provides OPSCC cells with the necessary energy for a rapid adaptation to the tumor microenvironment [62].

### 2.4. miR-31-5p

Upregulation of miR-31-5p has been observed in HNSCC cancers and has been confirmed by The Cancer Genome Atlas (TCGA) data analysis [63]. Functional assays have shown that miR-31-5p is an oncogenic miRNA in HNSCC [64]. Lai et al. reported that miR-31-5p overexpression leads to a marked increase in the free fatty acids accumulated, lipid droplet formation for cell proliferation, and migration in OSCC via targeting acyl-CoA oxidase 1 (ACOX1) [39]. ACOX1 is the first rate-limiting enzyme in the β-oxidation process of peroxisome fatty acids, and it is used to desaturate and oxidize linear lipid substrates, such as long-chain fatty acids and prostaglandins [65]. Prostaglandin E2 (PGE2) is one of the key substrates of peroxisome β-oxidation, and it is mediated by ACOX1. The expression level of PGE2 acts as a key mediator of miR-31-5p function, and it enhances cell motility by activating ERK-MMP9 signal transduction [39].

## 3. Exosomal miRNAs in HNSCC Tumor Microenvironment

**Figure 1:**
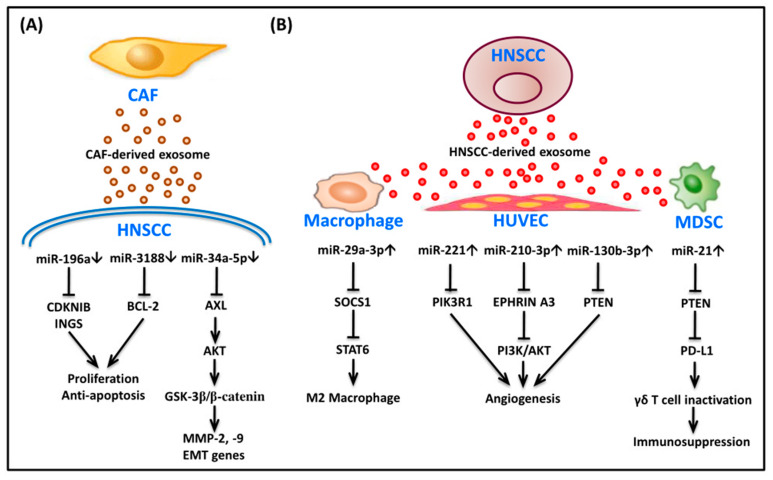
Summary of exosomal miRNAs and their key targets, including the associated signaling pathways involved in the HNSCC TME. (**A**) Exosomal miRNAs interact between CAF and HNSCC. The expression level of miR-196a, miR-3188, and miR-34a-5p in CAF-derived exosomes has been found to be significantly lower than that of NF-derived exosomes. These down-regulated miRNAs in CAF-derived exosomes modulate the migration, EMT, and anti-apoptosis, as well as tumor growth. (**B**) Exosomal miRNAs interact between HNSCC and HUVEC, and macrophage and MDSC. The differential expression of miRNAs in HNSCC-derived exosomes modulate the M2 polarization of microphage, the angiogenesis of HUVEC, and immunosuppression of MDSC in HNSCC TME. Abbreviations: BCL2, B-cell lymphoma 2; CAF, cancer- associated fibroblast; CDKN1B, cyclin dependent kinase inhibitor 1B; EMT, epithelial-mesenchymal transition; GSK-3β, Glycogen Synthase Kinase 3β; HNSCC, head and neck squamous cell carcinoma; HUVEC, human umbilical vein endothelial cell; ING5, inhibitor of growth family member 5; MDSC, myeloid-derived suppressor cell; MMP, matrix metallopeptidase; PD-L1, programmed death receptor ligand 1; PI3K, phosphoinositide 3-kinase; PIK3R1, phosphoinositide 3-kinase regulatory subunit 1; PTEN, phosphatase and tensin homolog deleted on chromosome 10; SOCS1, suppressor of cytokine signaling 1; STAT6, signal transduction and transcriptional activator 6; TME, tumor microenvironment.

The tumor microenvironment (TME) is composed of cancer cells and their surrounding spaces, and it is an intricate and interconnected system, consisting of the extracellular matrix (ECM) and different types of stromal cells [66]. The ECM comprises non-cellular components including collagen, laminin, fibronectin, elastin, glycoproteins, and proteoglycans, which are present in all tissue and provide an essential scaffolding for the cellular components [67]. The ECM also provides both structural and biomechanical support to modulate cellular function, such as cell–cell/or cell–matrix adhesion, cellular communication, and differentiation [68]. In addition to cancer cells, there are many different types of cells distributed in the ECM, including endothelial cells (ECs), cancer-associated fibroblasts (CAFs), and immune cells such as tumor-associated macrophages (TAMs), tumor-infiltrating lymphocytes (TILs), dendritic cells, etc. [29]. These cells are attracted to and collaborate with cancer cells for extracellular matrix remodeling and immune escape together with tumor progression and resistance to therapy [69]. Moreover, cancer and stromal cells are able to secrete soluble cytokines or signaling molecules into extracellular space to establish an advantageous ECM for cancer growth and metastasis [70]. Recently, exosomes have attracted growing interest from scientists, mainly because exosomes are used for cell-to-cell communication through endocrine, paracrine, and autocrine cells from cancer cells and a variety of stromal cells in the TME [71]. Exosomes, encased in lipid bilayers of cell membranes, are a class of extracellular nanovesicles, with a diameter range of 30–150 nm [72], that contain various endogenous cargoes, such as DNAs, mRNAs, non-coding RNAs, proteins, and lipids. In addition, exosomes possess specific surface proteins, including CD63, CD81, CD169, MHC/I/II, phosphatidylserine, integrins, etc. [73]. When exosomes are secreted to the surrounding and distant sites, they can bind to different types of cells through different receptors on the membrane, delivering these goods into the recipient cells via internalization [74]. According to reports, miRNA is one of the main cargoes in exosomes; it can control several biological processes that interact directly or indirectly between HNSCC cells and TME components [75]. Some highly metastatic or drug-resistant tumor cells can secrete exosomal miRNA to affect other low-metastatic or non-drug-resistant phenotype tumor cells, which are more suitable for their growth. For example, salivary exosomal miR-24-3p is able to promote tumor growth and regulate the expression of cell cycle-related genes by targeting PER1 in OSCC [76]. Another study performed by Liu et al. found that exosomes derived from cisplatin-resistant OSCC cells can transfer miR-21 to cancer cells, and by targeting phosphatase and tensin homolog (PTEN) and programmed Cell death 4 (PDCD4), can induce cisplatin resistance [77]. Otherwise, exosomal miRNAs, detectable in circulating body fluids, can be used as potential diagnostic and prognostic biomarkers for HNSCC [75,78]. For example, miR-21 has been reported to be highly expressed in serum-derived exosomes, and its expression has been found to be significantly correlated to the clinical outcomes of LSCC [79]. Along with HNSCC cells, other stromal cells in the TME can also take up exosomal miRNA from tumor cells and change their behavior, consequently creating an advantageous environment for tumor growth. In the next paragraphs, the oncogenic crosstalk, mediated by miRNAs, including exosomal miRNAs in different cell types of the TME is discussed. A summary of exosomal miRNAs and their key targets, including the associated signaling pathways involved in the HNSCC TME, are shown in Figure 1.

### 3.1. Exosomal miRNAs Crosstalk between HNSCC and TAMs

Macrophages, originally derived from peripheral blood monocytes, are among the most important immune cells within the tissues, responsible for innate and acquired immune responses to pathogens. It is known that most malignant tumors are able to recruit macrophages to surround the tumor microenvironment, and they function as a main constituent of the host immune infiltration [80]. These macrophages are called tumor-associated macrophages (TAMs), and under the stimulation of various secreted factors, they can polarize from an M1-like anti-tumor phenotype to an M2-like pro-tumor phenotype [81]. M1-like macrophages secrete many cytokines, such as interleukin (IL)-1β, IL-6, and tumor necrosis factor-α (TNF-α), to the surrounding environment, and these cytokines have pro-inflammatory effects and promote an immunity response to avoid carcinogenesis [82]. On the other hand, M2-like macrophages, which express a large amount of anti-inflammatory cytokines, chemokines, growth factors, and angiogenesis factors, are imperative for tumor development [83]. Importantly, high levels of TAMs are often related to a poor clinical outcome in solid tumors [84]. Cai et al. demonstrated that OSCC-derived exosomes could promote polarization of macrophages to the M2 phenotype. High levels of miR-29a-3p were found in OSCC-derived exosomes. The exosome-encapsulated miR-29a-3p is transferred to non-polarized macrophages, and the macrophages are then activated and polarized to the M2 phenotype through the activation of the suppressor of cytokine signaling 1 (SOCS1)/signal transduction and transcriptional activator 6 (STAT6) signals [85]. miR-550a-3p is another miRNA that has been reported to regulate M2 macrophage polarization. Its low expression is negatively mediated by oncoprotein E6 and is associated with human papillomavirus (HPV)-positive OSCC in metastasis. Cao et al. found that YAP, directly targeted by miR-550a-3-5p, can induce the expression and secretion of C-C motif chemokine ligand 2 (CCL2) into the TME, thereby promoting the polarization of M2 macrophages, and thereby inducing migration, invasion, and EMT in HPV-positive OSCC cells [86].

### 3.2. Exosomal miRNAs Crosstalk between HNSCC and TILs

TILs are considered the most crucial effectors to protect against tumor development in the TME [87]. Importantly, several studies have recognized TILs as an important biomarker and a favorable prognostic factor for treatment and clinical outcomes of HNSCC [88,89]. The major components of TILs are T lymphocytes, including cytotoxic T lymphocytes (CD8+ T cells), and helper T lymphocytes (CD4+ T cells), which play a role in tumor elimination [90,91]. Recently, B cells, another important component of TILs, have been found to be able to influence the progression of tumors through producing antibodies against the tumor antigen [92]. These TILs are activated by cancer cells, which present the tumor antigens, or by antigen-presenting lymphocytes, such as macrophages and dendritic cells (DCs) [93]. In addition, γδ T lymphocytes, express T cell receptor Vγ9 and Vγ2 chains on the cell membrane represent a minor CD3+ lymphocyte population in the peripheral blood [94]. It has been reported that γδ T lymphocytes have direct cytotoxicity to cancer cells by stimulating the expansion of cytotoxic effector T lymphocytes [95]. Li et al. have demonstrated that OSCC cell-derived exosomes containing miR-21 could regulate the cytotoxicity of γδ T lymphocytes under the hypoxia TME [96]. They demonstrated that normoxic exosomes, derived from OSCC cells, can stimulate the expansion and activation of γδ T lymphocytes, which rely on HSP70-mediated contact between tumor-derived exosomes and γδ T lymphocytes. On the other hand, hypoxic OSCC cells can activate the immunosuppressive function of myeloid-derived suppressor cells (MDSCs) through exosomes—which carry increased levels of miR-21—by targeting PTEN, thereby increasing programmed death receptor ligand 1 (PD- L1) expression. Since γδ T lymphocytes express PD-1, the combination of PD-1 and PD-L1 of MDSC induces γδ T lymphocyte depletion in a PD-L1/PD-1 dependent pattern.

### 3.3. Exosomal miRNAs Crosstalk between HNSCC and CAFs

Fibroblasts, derived from primitive mesenchymal cells, are the main cell type of connective tissue. Normal fibroblasts produce large amounts of extracellular matrix and collagens for maintaining the structural integrity of epithelium tissues and the host immune response [97]. CAFs, a major component of the tumor microenvironment, principally originate from the transformation of normal fibroblasts (NFs) and mesenchymal stem cells (MSCs) [98]. In addition, CAFs can also be derived from smooth muscle cells, epithelial cells, endothelial cells, or adipose-derived stem cells through different activation processes [98,99]. In the TME, CAFs have been shown to facilitate tumor survival, proliferation, angiogenesis, ECM remodeling, and immunosuppression through their secretion of several growth factors, cytokines, and chemokines [99,100]. Additionally, recent studies have reported that exosomal miRNAs, released into the TME by CAFs, can modulate the characteristics of the tumor cells. In OSCC, the expression level of miR-34a-5p in CAF-derived exosomes has been found to be significantly lower than that of NF-derived exosomes [101]. Downregulation of miR-34a-5p increases the expression level of AXL, an oncogenic receptor tyrosine kinase, which is the direct target of miR-34a-5p and mediates the proliferation and movement of OSCC cells through the AKT/GSK-3β/β-catenin signaling pathway, which could induce transcriptional upregulation of SNAIL to promote matrix metallopeptidase expression and EMT. Similar findings indicate that CAF-derived exosomes contain lower miR-3188 levels than NFs, which contributes to HNSCC malignancies [102]. The loss of miR-3188 can increase the expression level of B-cell lymphoma 2 (BCL2) in HNSCC cells, subsequently increasing cell proliferation and promoting the transition from G1 to the S cell cycle, as well as promoting cell apoptosis. In addition, drug resistance is a key factor affecting the prognosis of patients and has thus been receiving increasing attention. Qin et al. revealed that the heterogeneous nuclear ribonucleoprotein A1 (hnRNPA1) protein mediates the packaging of miR-196a into CAF-derived exosomes [103]. This study shows that CAF-derived exosomal miR-196a has the ability to promote the cisplatin resistance of HNSCC by targeting cyclin dependent kinase inhibitor 1B (CDKN1B) and inhibitor of growth family member 5 (ING5), leading to cell proliferation and inhibiting cell apoptosis. Another interesting CAF-derived miRNA is miR-7, which causes overexpression in NFs and induces a functional change of NFs into CAFs in HNSCC. The upregulation of miR-7 has been found to decrease the expression of Ras association domain family member 2 (RASSF2), which consequently decreases prostate apoptosis response-4 (PAR-4) secretion by CAFs, thereby increasing the proliferation and migration of the HNSCC cells [104].

### 3.4. Exosomal miRNAs Crosstalk between HNSCC and ECs

All tissues within the body rely on a blood supply to provide the cells with nutrients and oxygen, and the blood supply depends on endothelial cells, which form the inner wall of the blood vessels. For the tumor microenvironment, remodeling of the ECs and formation of new vessels (angiogenesis) is essential for tumor growth, development, and metastasis [105]. ECs have the extraordinary ability to adjust their number and arrangement to increase the density of microvessels, which are stimulated by a complex network of signaling pathways and soluble factors [106]. To date, numerous studies have shown that molecules delivered by exosomes circulate freely throughout body fluids and accumulate in the TME, playing an important role in regulating tumor angiogenesis [107,108], even in HNSCC [109]. Among them, miRNAs are a group of exosomal shuttle molecules that are essential factors used by tumors for reprogramming of endothelial cells [109]. A study showed that miR-210-3p was overexpressed in OSCC tissues and correlated with microvessel density and tumor grade [110]. It has been reported that microvessel density reflects the intensity of HNSCC angiogenesis [111]. More precisely, miR-210-3p has been found to be expressed by OSCC cells and secreted to human umbilical vein endothelial cells (HUVECs) through exosomes. In HUVEC cells, ephrin A3 is targeted and downregulated by miR-210-3p, and it promotes tube formation through the PI3K/AKT signaling pathway [110]. Another study reported that the exosomal miR-221, secreted by OSCC cells, inhibits the functional role of phosphoinositide 3-kinase regulatory subunit 1 (PIK3R1) in regulating HUVEC cell migration and tube formation, leading to angiogenesis [112]. Recently, Yan et al. also indicated that exosome-miR-130b-3p is a promoter of OSCC angiogenesis, which enhances the progression and tubular formation of HUVEC in vitro and in vivo. Moreover, the effect of exosomal miR-130b-3p on angiogenesis works by directly targeting the phosphatase and tensin homolog deleted on chromosome 10 (PTEN) [113].

## 4. Clinical Implications of miRNA in HNSCC

The advantageous features of miRNA, such as their short sequence, can protect them from degradation by endogenous RNase. Therefore, they are more stable in tissues (including fresh tissues and formalin-fixed paraffin-embedded tissues) or body fluids (such as blood, saliva, urine, and milk), making them an important diagnostic and prognostic biomarker for malignant tumors [9]. Recent studies have used miRNA expression profiles to define the diagnostic value of miRNA in HNSCC [10]. For example, Salazar-Ruales et al. comprehensively identified 4 miRNAs from 108 saliva samples of HNSCC patients and 108 controls using a PCR arrays technique to determine whether these miRNAs can be useful as HNSCC diagnostic biomarkers [114]. These four different miRNAs—miR-122-5p, miR-92a-3p, miR-124-3p, and miR-146a-5p—showed significant statistical difference of the area under the curve (AUC) values of 0.73, 0.70, 0.71, and 0.66, respectively, between the case group and control group, indicating that these saliva miRNAs could be promising biomarkers for HNSCC diagnosis. Another study evaluated the diagnostic potential of miR-196a and miR-196b in plasma samples, including 53 cases from healthy controls and 90 cases from oral cancer patients [115]. The ROC curves obtained, which showed obvious discrimination between normal and cancer patients (AUC = 0.864 and 0.960 for miR-196a and miR-196b, respectively) suggested that these miRNAs are specific circulating miRNA biomarkers for oral cancer patients when compared with the healthy controls. Otherwise, exosomal miRNAs can be identified in the TME and can circulate freely in body fluids, which also makes them suitable for acting as non-invasive biomarkers in clinical applications [116,117,118]. Utilizing a new generation of miRNA sequencing technology, Langevin et al. demonstrated that exosomal miR-486-5p, miR-486-3p, and miR-10b-5p were detectable in saliva for a set of HNSCC patients, and these might be exploited as non-invasive salivary biomarkers [119]. Another study by Gai and his colleagues investigated the saliva miRNA profile present in OSCC-derived extracellular vesicles [120]. They found that miR-302b-3p and miR-517b-3p were upregulated in the salivary extracellular vesicles of OSCC patients. Compared with the control group, the obtained AUC values were 0.847 and 0.87, respectively, showing a significant discrimination power for OSCC diagnosis.

Increasing evidence for the role of miRNAs in cancer development and progression highlights the clinical potential of miRNA as a therapeutic target. Based on the different functions of miRNAs, therapeutic miRNA approaches can be divided into miRNA mimics, to restore tumor-suppressive miRNA functions, and anti-miRs to inhibit tumor-promoting miRNA functions [121]. The miRNA mimic approaches mainly use synthetic double-stranded small RNA molecules and viral-associated vectors for restoring tumor-suppressive miRNAs [122]. The anti-miRNA approaches use antisense oligonucleotides, miRNA sponges, and miRNA masking for inhibiting tumor-promoting miRNAs [123]. Among these treatment strategies, locked nucleic acid (LNA) miRNA technology is the most commonly used, because of its high target affinity and low off-target toxicity [124]. It has been reported that antisense LNA-miR-24 and LNA miR-146a can inhibit cell growth and inhibit the carcinogenicity of oral cancer cells [125,126]. Although recent studies have shown promising results for miRNA-based treatments, such as miR-187-5p mimic and antagomiR-31-5p in oral cancer [127,128], there are currently no miRNA-targeting drugs approved, and no phase III trial has entered a clinical study so far [129]. In addition to the instability of RNA fragments and low transfection efficiency, the main challenge is designing efficient miRNA-targeting drugs carriers and delivery systems [130]. To date, miR-34 is the only miRNA in clinical trials (MRX34, from Mirna Therapeutics) [20]; however, this highlights the possibility of identifying miRNA targets as a new treatment technology to control HNSCC development.

Another notable therapeutic approach is exosome-based therapy, as the possible application of exosomes as therapeutic agents has recently become a point of research interest [131]. One of the therapeutic exosome strategies involves their use as carriers for the transport of antagonist tumor-suppressive miRNAs to cancer cells. Using cell-specific proteins present in the exosomal membrane, these encapsulated miRNAs can be injected systemically, or locally, into specific cancer cells [132]. Another therapeutic strategy involves inhibiting exosome release from the cancer cells or preventing uptake of exosomes by recipient cells. GW4869 is a neutral sphingomyelinase inhibitor, and it is the most commonly used drug to block the biogenesis and release of exosomes [133]. Dynasore is a dynein inhibitor that has been used to effectively block the endocytosis of exosomes or to inhibit their uptake [134]. Recent studies have demonstrated that exosome inhibitors can inhibit HNSCC cell growth and metastasis [135,136]. Thus, it could be possible to inhibit exosome transport and decrease exosome secretion from cancer cells to neighboring cells, which may lead to new targets for anticancer therapies.

## 5. Conclusions

In summary, miRNA research is an attractive, developing field, with miRNAs’ advantages based on their diversity for multiple targets, as well as their important role in tumor progression. These unique miRNAs not only participate in metabolic reprogramming regulation, but they are also involved in cell–cell communications in the TME. In particular, exosomal miRNAs’ ability to stably circulate in body fluids makes them a promising noninvasive liquid biomarker in HNSCC. The challenge in the future is to identify and enter more miRNAs with therapeutic potential into clinical trials. Due to the crucial role of miRNAs in HNSCC development, more research approaches are needed to explore the potential applications of miRNAs in cancer treatment.

## Figures and Tables

**Table 1:** miRNAs involved in HNSCC metabolism.

miRNA	Cancer Subtype	Application	Reference
miR-218	OSCC	Downregulation of miRNA-218 increases proliferation and glucose uptake by activating GLUT1 expression	[33]
miR-340	OSCC	Suppression of miRNA-340 activates GLUT1 expression, inducing proliferation, colony formation, lactate secretion, and glucose uptake rate	[34]
miR-10a	OSCC	miR-10a overexpression promotes glucose uptake and cell proliferation	[35]
miR-143	OSCC	Overexpression of miR-143 inhibited proliferation, migration, invasion, and glucose metabolism through targeting HK2	[36]
miR-125b-5p	LSCC	miR-125b-5p transfection decreased the glucose consumption and lactate production by targeting HK2, which inhibits cells growth	[37]
miR-210	OPSCC	HIF-1α-induced miR-210 silence ISCU expression, which promotes a switch from mitochondrial respiration to glycolytic metabolism	[38]
miR-31-5p	OSCC	miR-31-5p suppresses ACOX1 expression, enhancing MMP-9 and PGE2, promoting migration and invasion	[39]

OSCC: oral squamous cell carcinoma, LSCC: laryngeal squamous cell carcinoma, OPSCC: oropharyngeal squamous cell carcinomas.
